# Platelet-Released Growth Factors Influence Wound Healing-Associated Genes in Human Keratinocytes and Ex Vivo Skin Explants

**DOI:** 10.3390/ijms23052827

**Published:** 2022-03-04

**Authors:** Michael Singh, Serhat Akkaya, Mark Preuß, Franziska Rademacher, Mersedeh Tohidnezhad, Yusuke Kubo, Peter Behrendt, Jan-Tobias Weitkamp, Thilo Wedel, Ralph Lucius, Regine Gläser, Jürgen Harder, Andreas Bayer

**Affiliations:** 1 Institute of Anatomy, Kiel University, 24098 Kiel, Germany; m.singh@anat.uni-kiel.de (M.S.); serhat491@hotmail.de (S.A.); t.wedel@anat.uni-kiel.de (T.W.); rlucius@anat.uni-kiel.de (R.L.); 2 Department for Vascular Medicine, Heart and Vascular Center, University Medical Center Hamburg-Eppendorf, 20246 Hamburg, Germany; ma.preuss@uke.de; 3 Department of Dermatology, Venerology and Allergology, Kiel University, 24105 Kiel, Germany; frademacher@dermatology.uni-kiel.de (F.R.); rglaeser@dermatology.uni-kiel.de (R.G.); jharder@dermatology.uni-kiel.de (J.H.); 4 Department of Anatomy and Cell Biology, RWTH Aachen University, 52074 Aachen, Germany; mtohidnezhad@ukaachen.de (M.T.); ykubo@ukaachen.de (Y.K.); 5 Department of Trauma Surgery, University Medical Center of Schleswig-Holstein, Campus Kiel, 24105 Kiel, Germany; peter.behrendt.kiel@gmail.com; 6 Department of Oral and Maxillofacial Surgery, University Medical Center of Schleswig-Holstein, Campus Kiel, 24015 Kiel, Germany; jan-tobias.weitkamp@uksh.de

**Keywords:** platelet-released growth factors (PRGFs), keratinocytes, ex vivo skin explants, wound healing

## Abstract

Platelet-released growth factors (PRGFs) or other thrombocyte concentrate products, e.g., Platelet-Rich Fibrin (PRF), have become efficient tools of regenerative medicine in many medical disciplines. In the context of wound healing, it has been demonstrated that treatment of chronic or complicated wounds with PRGF or PRF improves wound healing in the majority of treated patients. Nevertheless, the underlying cellular and molecular mechanism are still poorly understood. Therefore, we aimed to analyze if PRGF-treatment of human keratinocytes caused the induction of genes encoding paracrine factors associated with successful wound healing. The investigated genes were Semaphorin 7A (SEMA7A), Angiopoietin-like 4 (ANGPLT4), Fibroblast Growth Factor-2 (FGF-2), Interleukin-32 (IL-32), the CC-chemokine-ligand 20 (CCL20), the matrix-metalloproteinase-2 (MMP-2), the chemokine C-X-C motif chemokine ligand 10 (CXCL10) and the subunit B of the Platelet-Derived Growth Factor (PDGFB). We observed a significant gene induction of SEMA7A, ANGPLT4, FGF-2, IL-32, MMP-2 and PDGFB in human keratinocytes after PRGF treatment. The CCL20- and CXCL10 gene expressions were significantly inhibited by PRGF therapy. Signal transduction analyses revealed that the PRGF-mediated gene induction of SEMA7A, ANGPLT4, IL-32 and MMP-2 in human keratinocytes was transduced via the IL-6 receptor pathway. In contrast, EGF receptor signaling was not involved in the PRGF-mediated gene expression of analyzed genes in human keratinocytes. Additionally, treatment of ex vivo skin explants with PRGF confirmed a significant gene induction of SEMA7A, ANGPLT4, MMP-2 and PDGFB. Taken together, these results describe a new mechanism that could be responsible for the beneficial wound healing properties of PRGF or related thrombocytes concentrate products such as PRF.

## 1. Introduction

Chronic or complicated wounds still display a major problem for the individual patient concerned and the health care system in general [1,2,3,4]. One emerging and promising therapeutic option is topical wound therapy with highly concentrated thrombocyte products, e.g., Platelet-released growth factors (PRGFs) or Platelet-Rich Fibrin (PRF) [5,6]. As these thrombocyte concentrate products contain a variety of growth factors, cytokines and chemokines [7,8], they are supposed to improve regenerative processes in the human body in general and wound healing in particular [9]. Although it has been demonstrated that the majority of wound patients benefit from the topical application of PRGF and PRF on their wounds [10,11], the underlying mechanisms are still poorly understood. Recently, we have shown that treatment of keratinocytes with PRGF caused induction of several antimicrobial peptides in the treated keratinocytes indicating the PRGF-mediated strengthening of the epidermal barrier function [12,13,14]. Moreover, PRGF treatment of keratinocytes and fibroblasts led to an accelerated epidermal hornification and an improved extracellular matrix synthesis [15,16,17]. As wound healing is a well-orchestrated and multicellular process [18,19], we now asked if PRGF treatment of keratinocytes causes the induction of factors that may act in a paracrine way on other cells, such as fibroblasts, endothelial cells and macrophages, that promote the wound-healing process. In this context, we analyzed the influence of PRGF on the gene expression of Semaphorin 7A (SEMA7A), Angiopoietin-like 4 (ANGPLT4), Fibroblast Growth Factor-2 (FGF-2), Interleukin-32 (IL-32), the CC-chemokine-ligand 20 (CCL20), the matrix-metalloproteinase-2 (MMP-2), the chemokine C-X-C motif chemokine ligand 10 (CXCL10) and the subunit B of the Platelet-Derived Growth Factor (PDFGB) in primary human keratinocytes. Moreover, we assessed the relevance of the Epidermal-derived Growth Factor Receptor (EGFR) and the Interleukin-6 (IL-6) pathway for the PRGF-mediated induction of the analyzed genes.

## 2. Results

### 2.1. Concentration-Dependent Influence of PRGF Treatment on the Expression of Wound-Healing-Associated Genes in Primary Normal Human Epidermal Keratinocytes (NHEKs)

**Figure 1:**
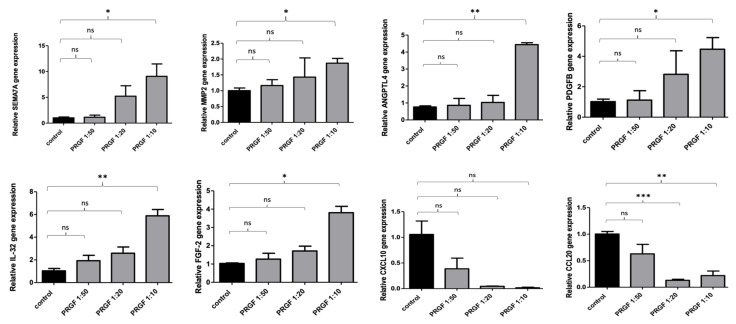
Concentration-dependent influence of PRGFs on the expression of wound-healing-associated genes in primary normal human epidermal keratinocytes (NHEKs). Primary human keratinocytes were stimulated for 24 h with PRGF in different concentrations. Relative gene expression was analyzed by real-time PCR. Shown are means ± s.e.m of three stimulations (* *p* < 0.05, ** *p* < 0.01, *** *p* < 0.001, ns = non-significant; Student’s *t*-test).

To investigate a possible influence on SEMA7A, MMP-2, ANGPLT4, PDGFB, IL-32, FGF-2, CXCL10 and CCL20 gene expression, keratinocytes were stimulated with different concentrations of PRGF (1:50, 1:20, 1:10) for 24 h. PRGF stimulation caused a significant increase in SEMA7A, MMP-2, ANGPLT4, PDGFB, IL-32 and FGF-2 gene expression paralleled by a significant decrease in CXCL10 and CCL20 gene expression in the stimulated keratinocytes (see Figure 1).

### 2.2. Time-Dependent Influence of PRGF Treatment on the Expression of Wound-Healing-Associated Genes in Primary Normal Human Epidermal Keratinocytes (NHEKs)

**Figure 2:**
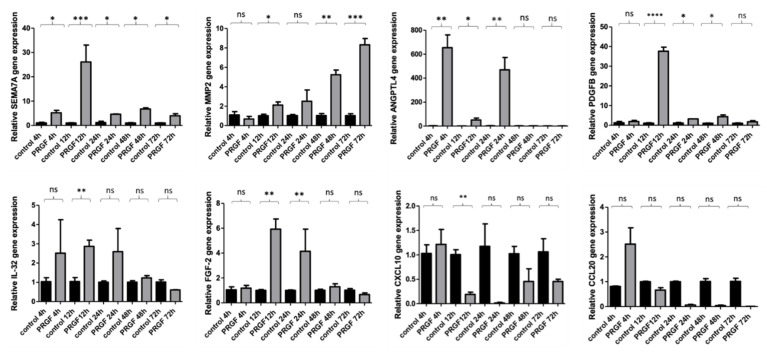
Time kinetics study from 4–72 h of PRGF-induced genes in primary normal human epidermal keratinocytes (NHEKs). Human primary keratinocytes were stimulated with PRGF (1:10) for the indicated periods. Relative gene expression was analyzed by real-time PCR. Shown are means ± s.e.m of three independent stimulations (* *p* < 0.05, ** *p* < 0.01, *** *p* < 0.001, **** *p* < 0.0001; ns = non-significant; Student’s *t*-test).

A time kinetic study from 4 to 72 h revealed a significant PRGF-associated gene expression of almost all investigated genes after 1–3 days incubation time. Except for CCL20 and CXCL10, all genes showed a significant induction at least after 12 h. SEMA7A and ANGPLT4 were significantly induced by PRGF already after 4 h (see Figure 2).

### 2.3. The Influence of the Epidermal Growth Factor Receptor (EGFR) Pathway on the PRGF-Mediated Expression of Wound Healing-Associated Genes in Human Keratinocytes

**Figure 3:**
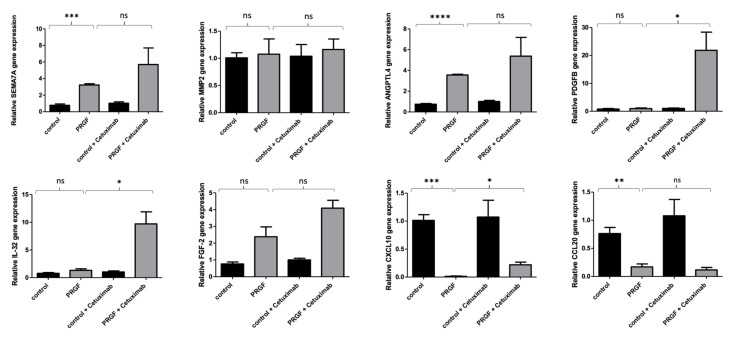
The EGFR influences the PRGF-mediated gene expression of PDGFB, IL-32 and CXCL10 in human keratinocytes. Human primary keratinocytes were stimulated for 24 h with PRGF (1:10) in the presence or absence of the EGFR blocking antibody cetuximab (20 µg/mL). Relative gene expression was analyzed by real-time PCR. Shown are means ± s.e.m of three independent stimulations (* *p* < 0.05, ** *p* < 0.01, *** *p* < 0.001, **** *p* < 0.0001; ns = non-significant, Student’s *t*-test).

In previous studies, we observed a relevant influence of the EGFR on the PRGF-mediated induction of antimicrobial peptides and ECM-related factors in keratinocytes. Therefore, we now aimed to analyze the influence of the EGFR on the observed PRGF-mediated influence of wound healing-associated genes in human keratinocytes. Therefore, we used the monoclonal EGFR-antibody cetuximab to block and inactivate signal transduction by the EGFR. These experiments revealed that the PRGF-mediated gene inductions described above were not dependent on the EGFR pathway. The blockade of the EGFR by cetuximab caused a significant gene induction of PDGFB, IL-32 and CXCL10 in human keratinocytes (Figure 3).

### 2.4. The Influence of the IL-6 Receptor Pathway on the PRGF-Mediated Expression of Wound-Healing-Associated Genes in Human Keratinocytes

**Figure 4:**
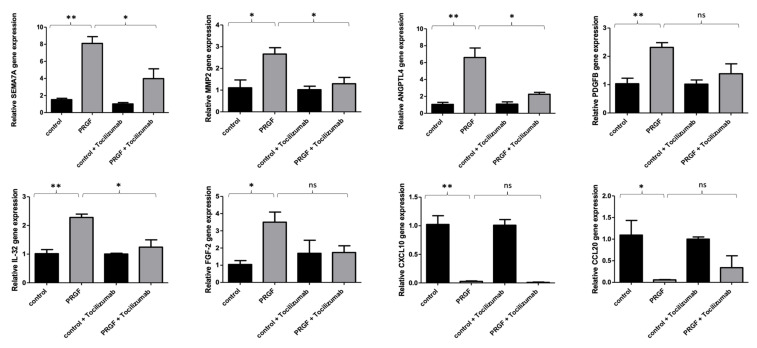
The PRGF-mediated gene induction of SEMA7A, MMP-2, ANGPLT4 and IL-32 in human keratinocytes is transduced via the IL-6 receptor. Human primary keratinocytes were stimulated for 24 h with PRGF (1:10) in the presence or absence of the IL-6-receptor blocking antibody tocilizumab 50 µg/mL). Relative gene expression was analyzed by real-time PCR. Shown are means ± s.e.m of three stimulations (* *p* < 0.05, ** *p* < 0.01; ns = non-significant, Student’s *t*-test).

To examine the underlying signal transduction pathways of the PRGF-mediated SEMA7A, MMP-2, ANGPLT4, PDGFB and IL-32 gene induction in primary human keratinocytes, we analyzed the influence of the interleukin-6 receptor (IL-6R) using a specific monoclonal IL-6R-blocking antibody (tocilizumab). The blockade of the IL-6R by tocilizumab caused a significant inhibition of the PRGF-mediated gene induction of SEMA7A, MMP-2, ANGPLT4 and IL-32 in human keratinocytes. The PRGF-mediated gene induction of PDGFB in human keratinocytes was not transduced via the IL-6 pathway. The decreased gene expression of CXCL10 and CCL20 in PRGF-treated keratinocytes was independent of IL-6 signaling (see Figure 4).

### 2.5. PRGF Treatment Caused Induction of Wound-Healing-Associated Genes in Human Ex Vivo Skin Explants

**Figure 5:**
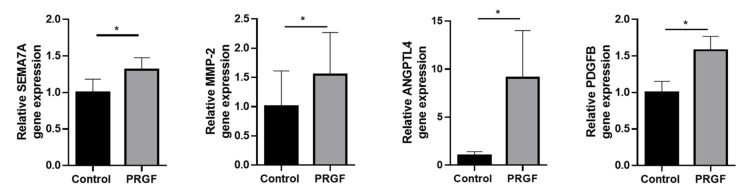
PRGF-mediated gene expressions in ex vivo skin explants. Human ex vivo skin explants were incubated with PRGF (1:5 diluted in phosphate-buffered saline) for 24 h. Relative gene expression was analyzed by real-time PCR. Shown are means ± s.e.m of *n* = 18 (Control) and *n* = 20 (PRGF) stimulations (* *p* < 0.05, Mann–Whitney test).

In order to investigate whether we can confirm the described in vitro effects in the ex vivo setting, we stimulated human ex vivo skin explants with PRGF. These experiments revealed a significant gene induction of SEMA7A, MMP-2, ANGPLT4 and PDGFB in human ex vivo skin explants (see Figure 5).

## 3. Discussion

Autologous platelet concentrates, such as PRGF or PRF, contain a variety of chemokines, cytokines and growth factors [7,20,21]. Therefore, they have been used in many medical disciplines in recent years to generally optimize tissue regeneration [22,23]. Against this background, autologous platelet concentrates also represent a promising therapeutic option for the treatment of chronic or complicated wounds [10,11,24]. The underlying mechanisms for the positive clinical wound healing processes have not yet been adequately clarified. Cell culture experiments have shown that the treatment of human keratinocytes with autologous platelet concentrates such as PRGF led to an induction of antimicrobial peptides in the keratinocytes and thus to an optimization of the epithelial barrier function [12,13,14]. In addition, the treatment of keratinocytes with PRGF appears to accelerate epithelial keratinization [15] and improve extracellular matrix formation [16,17]. The EGF and IL-6 receptors seem to play an essential role in the signal transduction of these effects [12,13,14].

The aim of the present study was to investigate whether the treatment of primary human keratinocytes with the platelet concentrate PRGF leads to an induction of genes encoding factors that in turn may activate other cells involved in wound healing, such as fibroblasts, endothelial cells and/or macrophages. This is important because wound healing is a highly complex and well-orchestrated process involving not only keratinocytes but also, among others, the cells mentioned above [25,26,27]. Additionally, the role of the EGF and IL-6 receptors in these signal transduction pathways were investigated as they are involved in the aforementioned effects.

SEMA7A, also known as CD108, is a membrane-bound semaphorin that associates with cell surfaces via glycosylphosphatidylinositol binding and generally has proinflammatory effects [28,29,30]. In addition, SEMA7A appears to have a positive influence on wound healing [31]. At the cellular level, it has been shown that the expression of SEMA7A on keratinocytes leads to the activation of resident macrophages in the context of wound healing [32]. In our experiments, we observed a significant SEMA7A gene induction in PRGF-treated keratinocytes after four to 72 h of stimulation. This effect was not dependent on the EGF receptor but was dependent on the IL-6 receptor. Thus, SEMA7A regulation in keratinocytes—at least in this experimental setting—seems to occur via a different signal transduction pathway than, for example, in malignant cells [33].

Angiopoietin-like 4 is a multifunctional protein that may have various functions in the context of tumorigenesis, angiogenesis, inflammation and stem cell regulation [34,35,36]. In the context of wound healing, ANGPLT4 activates specific integrins that are important for successful wound healing [37]. In cell culture experiments, there is evidence that ANGPLT4 can lead to the recruitment and activation of various immune cells into the wound, thereby improving wound healing [38]. Furthermore, ANGPLT4 plays a role in the regulation of various cell–matrix interactions and cell migrations that are important for effective wound healing [39,40,41]. ANGPTL4 produced by keratinocytes interacts with the fibroblasts-derived extracellular matrix proteins vitronectin and fibronectin in the wound bed, thereby delaying their proteolytic degradation by metalloproteinases [39]. Moreover, ANGPLT 4 appears to play a role in the cellular crosstalk of keratinocytes and endothelial cells in the context of angiogenesis in wound healing, thereby positively influencing it [41,42]. We observed that treatment of human keratinocytes with PRGF resulted in a high induction of ANGPLT4 already after a 4 h stimulation period, indicating a direct and rapid induction of ANGPLT4 by PRGF. After 12 h, the induction level declined and increased after 24 h. The induction after 24 h was inhibited by blocking the IL-6R, suggesting that a paracrine indirect stimulation via secreted IL-6 takes place.

FGF-2, also known as basic fibroblast growth factor (b-FGF), is also a multifunctional growth factor [43] that can positively influence wound healing [44,45,46]. Various cellular and molecular mechanisms have been described in this context. FGF-2 is known for its proliferation-promoting activity on different cell types involved in wound healing, such as fibroblasts and keratinocytes [47]. PRGF-mediated FGF-2 gene induction in human keratinocytes was significant in our experiments after 12 and 24 h of stimulation. The PRGF-mediated induction was reduced in the presence of the IL-6 receptor blocking antibody tocilizumab, suggesting that IL-6 signaling is involved in this process. This is in line with our previous study documenting a rapid PRGF-mediated induction of IL-6 in keratinocytes already after 4 h [12].

IL-32 is a proinflammatory cytokine expressed on monocytes, endothelial and epithelial cells, among others [48,49]. It functions as a mediator and effector of the immune response by inducing various chemokines and proinflammatory cytokines, such as IL-1ß, IL-6, IL-8, TNF-alpha and macrophage inflammatory protein-2 (MIP-2) [50]. In vivo IL-32 is able to improve the healing of infected wounds [51]. In our experiments, treatment of human keratinocytes with PRGF resulted in a significant IL-6 receptor-dependent IL-32 gene induction after 12 and 24 h of PRGF treatment.

CC chemokine ligand-20 (CCL20) or Macrophage Inflammatory Protein-3A (MIP3A) is a protein of the CC chemokine family. CCL20 is induced in keratinocytes as a result of wounding [52] and has chemotactic effects on lymphocytes and neutrophil granulocytes, among others [53]. CCL20 generally contributes to the immune response and supports healing of the skin [54] and various epithelia [55]. The expression of the CCL20 receptor C-C motif chemokine receptor 6 (CCR6) on immune cells also appears to play an essential role in wound healing [56]. In contrast, CCL20 could also negatively influence wound healing by the inhibition of keratinocyte migration [57]. In our experiments, PRGF treatment of keratinocytes inhibited CCL20 gene expression. The EGF and IL-6 receptors did not play a role in the PRGF-mediated regulation of CCL20 in human keratinocytes.

C-X-C chemokine ligand-10 (CXCL10) is a protein in the chemokine group that is a surface protein involved in inflammation, tumor metastasis, angiogenesis and wound healing [58,59,60,61]. CXCL10 binds its receptor CXCR3 (CXC motif chemokine receptor 3) [62]. CXCL10 and its receptor CXCR3 play a role in the pathogenesis of many autoimmune diseases, and both proteins are expressed on various cells [63]. In the mouse model, it has been shown that reduced expression of CXCL10 may be associated with reduced angiogenesis and vascular integrity, among other factors in the wound [64]. In addition, CXCL10 expression was directly associated with accelerated wound healing in the mouse model [65]. The importance of CXCL10 in wound healing was further underlined by a delayed and pathological wound healing process in CXCR3 knock-out mice [66]. Furthermore, it has been described in the porcine in vivo model that the activation of the CXCL10/CXCR3 ligand-receptor cascade in macrophages can stimulate tissue regeneration [67]. In addition, the CXCR3 signaling cascade appears to be necessary for the physiological function of fibroblasts in the wound [68]. On the contrary, CXCL10 gene expression was associated with a decreased angiogenesis and delayed wound healing process [69,70]. Therefore, CXCL10 seems to exert a beneficial as well as a negative impact on the wound-healing process. In our experiments, we observed an inhibition of CXCL10 gene expression in PRGF-treated keratinocytes, which was independent of the IL-6 receptor. A reduced inhibition was observed by the EGF receptor blockade, suggesting that factors responsible for the PRGF-mediated CXCL10 inhibition may partly activate the EGFR signaling pathway.

Matrix metalloproteinase-2 (MMP-2) is a collagenase that plays a role in tissue regeneration [71,72], angiogenesis [73,74], inflammation [75,76], tumor metastasis [77] and wound healing [78,79]. In the context of wound healing, MMP-2 expression in keratinocytes was shown to directly accelerate the migration of these cells [80]. This effect also appeared to be directly associated with accelerated wound healing [81]. MMP-2 also promoted the wound-healing abilities of fibroblasts. In the context of the complex wound-healing process, it is essential that the activity of this collagenase is well dosed. It has been shown that excessive activity of MMP-2 can also lead to inhibition of wound healing [82,83]. The observed MMP-2 induction in PRGF-treated keratinocytes in most of our experiments may contribute to the beneficial effects of PRGF on the wound-healing process.

Platelet-derived growth factor subunit B (PDGFB) is a multifunctional growth factor that has an important function in the regulation of angiogenesis, inflammation, cell proliferation and migration [84,85,86] and thus also in wound healing [87,88]. Thereby, the expression of PDFGB in the wound seems to promote wound healing in vivo [89]. For this reason, there have been multiple clinical approaches to the direct therapeutic use of recombinant PDGFB in vivo [90,91]. The molecular and cellular basis for the underlying effects of the hoped-for positive clinical wound-healing processes under recombinant PDGF wound therapy is supposed to be an accelerated proliferation of keratinocytes and endothelial cells [92]. In our experiments, we found a significant gene induction of PDGFB in PRGF-stimulated keratinocytes after a stimulation period of 12 to 48 h, which was not transduced via IL-6 and EGF receptor activation.

In summary, we demonstrated in our cell culture experiments that the treatment of primary human keratinocytes with platelet-released growth factor (PRGF) leads to an induction of SEMA7A, ANGPLT4, FGF-2, IL-32, MMP2 and PDGFB. As seen for the PRGF-mediated induction of antimicrobial peptides [12,13,14], this induction partly depended on the IL-6-receptor pathway. This suggests that the rapid PRGF-mediated IL-6 induction already after 4 h [12] promotes an IL-6 dependent paracrine induction of several factors in PRGF-treated keratinocytes. The known beneficial effects of the above-mentioned factors in the wound-healing process may contribute to the observed positive clinical effects of thrombocyte-derived formulations to support wound healing. Remarkably, the inhibition of the EGFR promotes the PRGF-mediated induction of some factors, such as PDGFB and IL-32. This suggests that PRGFs contain factor(s) that activate the EGFR, thereby controlling the enhanced expression of PRGF-induced wound-healing mediators. This may avoid an excessive PRGF-mediated accumulation of these factors that otherwise may have rather detrimental consequences for the wound-healing process.

CXCL10 and CCL20 gene expressions in human keratinocytes were inhibited by PRGF treatment. Since wound healing-promoting and inhibitory effects of these factors have been described, the PRGF-mediated inhibition of these factors during specific steps of wound healing may also exert beneficial effects.

The observed PRGF-mediated gene regulations of the investigated factors in human keratinocytes may have an important influence on the function and activity of macrophages, fibroblasts, keratinocytes, endothelial cells and various immune cells during the wound-healing process. As successful wound healing depicts a complex, well-orchestrated and multicellular process, these new observations may help to explain the positive clinical wound-healing effects under a topical wound therapy with platelet concentrates such as PRGF or PRF.

## 4. Material and Methods

### 4.1. Preparation of PRGF

PRGF preparation was performed from freshly donated human thrombocyte concentrates by centrifugation, ultrasound treatment and repeated freezing and thawing, as described before [12].

### 4.2. Cell Culture and Stimulation of Primary Human Keratinocytes

Primary normal human epidermal keratinocytes (NHEKs) pooled from different individuals were purchased from Promocell and cultured in Keratinocyte Growth Medium 2 (KGM-2, Promocell, Heidelberg, Germany) at 37 °C in a humidified atmosphere with 5% CO_2_. For stimulation, we seeded the NHEKs in 12-well tissue culture plates (BD Biosciences, Franklin Lakes, NJ, USA). Cells were stimulated at a confluence of 80–100% with the indicated dilutions of the PRGF for the indicated time periods. To assess the influence of the EGFR on the investigated genes, we used the EGFR-blocking antibody cetuximab (20 µg/mL; Merck, Darmstadt, Germany). The relevance of the IL-6 pathway was assessed with the usage of the IL-6 receptor blocking antibody tocilizumab (50 µg/mL; Hoffmann-La Roche, Basel, Switzerland).

### 4.3. RNA Isolation and cDNA Synthesis

RNA isolation and cDNA synthesis were performed as described before [13]. In summary, NHEKs from one well of a 12-well plate were harvested and lysed with 500 µL Crystal RNAmagic. Subsequently, total RNA was isolated according to the supplier’s protocol (Biolab-Products, Bebensee, Germany). RNA quantity and quality were assessed photometrically with a NanoDrop device (Peqlab, Erlangen, Germany). 1 µg of total RNA was reversely transcribed to cDNA using oligo-dT-primers and 50 Units Maxima Reverse Transcriptase according to the manufacturer’s protocol (Thermo Fisher Scientific, Waltham, MA, USA).

### 4.4. Quantitative Real-Time PCR

Quantitative real-time PCR analyses were performed in a fluorescence-temperature cycler (StepOne Plus, Life Technologies) as previously described [93]. We generated gene-specific standard curves using serial dilutions of cDNA. All quantifications were normalized to the house keeping gene RPL38 (ribosomal protein L38). We determined the relative gene expression as a ratio between the indicated gene and the RPL38 gene expressions. The intron spanning primers used for gene expression analyses are presented in Table 1.

### 4.5. Expression Analysis of Wound Healing-Associated Genes in Ex Vivo Skin Explants

In ex vivo experiments, skin explants were incubated with PRGF followed by analysis of gene expression as described recently [16]. Briefly, skin explants obtained as waste material from surgeries were cut into small defined pieces and incubated with PRGF diluted 1:5 in PBS for 24 h. Subsequently, total RNA was isolated and used for cDNA synthesis and real-time PCR, as described above.

## Figures and Tables

**Table 1:** Primer sequences used for gene expression analyses by quantitative real-time PCR.

Gene	Forward Primer	Reverse Primer
Semaphorin 7A (SEMA7)	GATACTGTCATGCAGAACCC	GTAGTAGATCTTGTCATCGTAAGC
Angiopoietin-like 4 (ANGPLT4)	GGGACGAGATGAATGTCCT	CTTGAGTTGTGTCTGCAGG
Fibroblast Growth Factor-2 (FGF-2)	GTTGTGTCTATCAAAGGAGTGTG	TCCGTAACACATTTAGAAGCCAG
Interleukin-32 (IL-32)	CGACTTCAAAGAGGGCTACC	GAGTGAGCTCTGGGTGCTG
CC-chemokine-ligand 20 (CCL20)	CCAAGAGTTTGCTCCTGGCT	TGCTTGCTGCTTCTGATTCG
Matrix-metalloproteinase-2 (MMP-2)	AGCGAGTGGATGCCGCCTTTAA	CATTCCAGGCATCTGCGATGAG
Chemokine C-X-C motif chemokine ligand 10 (CXCL10)	GGTGAGAAGAGATGTCTGAATCC	GTCCATCCTTGGAAGCACTGCA
Subunit B of the Platelet-Derived Growth Factor (PDFGB)	GAGATGCTGAGTGACCACTCGA	GTCATGTTCAGGTCCAACTCGG
Ribosomal protein L38 (RPL38)	TCAAGGACTTCCTGCTCACA	AAAGGTATCTGCTGCATCGAA
